# Reconstructed Human Skin with Hypodermis Shows Essential Role of Adipose Tissue in Skin Metabolism

**DOI:** 10.1007/s13770-023-00621-1

**Published:** 2024-02-17

**Authors:** Jonas Jäger, Irit Vahav, Maria Thon, Taco Waaijman, Bas Spanhaak, Michael de Kok, Ranjit K. Bhogal, Susan Gibbs, Jasper J. Koning

**Affiliations:** 1 Department of Molecular Cell Biology and Immunology, Amsterdam UMC Location Vrije Universiteit Amsterdam, De Boelelaan 1117, Amsterdam, The Netherlands; 2 Amsterdam Institute for Infection and Immunity, Inflammatory Diseases, Amsterdam, The Netherlands; 3 Amsterdam Movement Sciences, Tissue Function & Regeneration, Amsterdam, The Netherlands; 4 Systems Biology Lab, Amsterdam Institute of Molecular and Life Sciences (AIMMS), Vrije Universiteit Amsterdam, Amsterdam, The Netherlands; 5 Unilever R&D, Colworth Science Park, Bedford, UK; 6 Department of Oral Cell Biology, Academic Centre for Dentistry Amsterdam (ACTA), University of Amsterdam and Vrije Universiteit, Amsterdam, The Netherlands

**Keywords:** Organotypic models, Skin substitutes, Metabolism, Subcutaneous adipose tissue, Adipocytes

## Abstract

**Background:**

Dysregulation of skin metabolism is associated with a plethora of diseases such as psoriasis and dermatitis. Until now, reconstructed human skin (RhS) models lack the metabolic potential of native human skin, thereby limiting their relevance to study human healthy and diseased skin. We aimed to determine whether incorporation of an adipocyte-containing hypodermis into RhS improves its metabolic potential and to identify major metabolic pathways up-regulated in adipose-RhS.

**Methods:**

Primary human keratinocytes, fibroblasts and differentiated adipose-derived stromal cells were co-cultured in a collagen/fibrin scaffold to create an adipose-RhS. The model was extensively characterized structurally in two- and three-dimensions, by cytokine secretion and RNA-sequencing for metabolic enzyme expression.

**Results:**

Adipose-RhS showed increased secretion of adipokines. Both RhS and adipose-RhS expressed 29 of 35 metabolic genes expressed in *ex vivo* native human skin. Addition of the adipose layer resulted in up-regulation of 286 genes in the dermal-adipose fraction of which 7 were involved in phase I (CYP19A1, CYP4F22, CYP3A5, ALDH3B2, EPHX3) and phase II (SULT2B1, GPX3) metabolism. Vitamin A, D and carotenoid metabolic pathways were enriched. Additionally, pro-inflammatory (IL-1β, IL-18, IL-23, IL-33, IFN-α2, TNF-α) and anti-inflammatory cytokine (IL-10, IL-12p70) secretion was reduced in adipose-RhS.

**Conclusions:**

Adipose-RhS mimics healthy native human skin more closely than traditional RhS since it has a less inflamed phenotype and a higher metabolic activity, indicating the contribution of adipocytes to tissue homeostasis. Therefore it is better suited to study onset of skin diseases and the effect of xenobiotics.

**Supplementary Information:**

The online version contains supplementary material available at 10.1007/s13770-023-00621-1.

## Introduction

Forming the outermost layer of the human body, skin serves as a complex interface between internal organs and the environment. In addition to having a vital protective barrier function, skin is also a major metabolically active organ [1, 2]. Dysregulation of skin metabolism is associated with a plethora of diseases such as psoriasis, dermatitis and melanoma [3, 4]. The skin contains enzymes to metabolize endogenous substances such as hormones, lipids and carbohydrates as well as enzymes to metabolize xenobiotics such as foreign chemicals and drugs [5, 6]. Substances penetrating the outermost *stratum corneum* layer of the skin can become metabolized into harmful or harmless by-products. Therefore, pre-clinical efficacy testing of new drugs that target skin diseases as well as safety testing of their individual components is important. Traditionally, this is being done using animals. However, in addition to raising ethical questions, animal experiments often poorly predict human responses [7]. Reconstructed human epidermis (RhE) and skin (RhS) are three-dimensional (3D) *in vitro* models mimicking the human skin and offer a promising solution for pre-clinical efficacy testing and safety assessment of compounds in an animal-free setting [8–12]. Current RhE and RhS lack complexity and do not include the subcutaneous adipose layer which is a metabolically very active skin layer [13]. Consequently, these models do not fully recapitulate the metabolic potential of human skin, thereby limiting their relevance for safety testing and to study human skin diseases [5, 6].

Several models integrating adipocytes into RhS have been described [14–19], but none of them specifically addresses the effect of the adipose layer on skin metabolism.

Therefore, the aim of this study was to determine whether the metabolic activity is increased in a tri-layered RhS model consisting of a: (1) stratified and differentiated epidermis, (2) fibroblast-populated collagen/fibrin hydrogel functioning as dermis and (3) differentiated adipose-derived stromal cells (ASCs) in a collagen/fibrin hydrogel functioning as hypodermis (adipose-RhS). First, the extent of ASC-differentiation towards adipocytes was determined before they were integrated into adipose-RhS. Next, RNA-sequencing (RNA-seq) was used to analyze phase I and phase II metabolic enzymes in RhS and adipose-RhS. Epidermis and dermis were screened for differentially expressed genes (DEGs) and analyzed for gene ontology (GO) pathways. In addition, the secretion of various cytokines and adipokines was investigated.

## Materials and methods

### Cell culture

#### Fibroblasts and keratinocytes

Dermal fibroblasts and keratinocytes (KCs) were isolated from human foreskin as described previously [20, 21]. KCs were amplified in DermaLife K medium (Lifeline Cell Technology, Frederick, MD) with 1% penicillin/streptomycin (Life Technologies Corporation, Grand Island, NY) on 0.5 µg/cm^2^ collagen IV (Sigma-Aldrich, St. Louis, MO) coated plates. Medium was switched to keratinocyte medium I (KCI) and DermaLife K medium (1:1) one day before using the cells for construction of the epidermis. KCI consists of Dulbecco's modified Eagle's medium (DMEM; Lonza, Basel, Switzerland) and Ham’s F12 (Corning, Corning, NY) in a ratio of 3:1, supplemented with 1% penicillin/streptomycin, 5% FetalClone III (HyClone, Logan, UT), 1 µM hydrocortisone (Sigma-Aldrich), 1 µM isoproterenol hydrochloride (Sigma-Aldrich) and 0.1 µM insulin (Sigma-Aldrich). Fibroblasts were amplified in fibroblast medium: DMEM supplemented with 1% penicillin/streptomycin and 5% FetalClone III. Medium for both cell types was exchanged every 3–4 days, KCs were cultured at 37 °C, 7.5% CO_2_ and fibroblasts at 37 °C, 5% CO_2_.

#### Adipose-derived stromal cells

The ASC fraction was isolated from fat attached to skin as described previously and grown in fibroblast medium [20]. Five days before construction of the adipose layer, adipocyte differentiation was induced by ASC differentiation medium [22] consisting of DMEM:Ham’s F12 1:1, supplemented with 1% penicillin/streptomycin, 33 µM biotin (Sigma-Aldrich), 17 µM d-pantothenic acid hemicalcium salt (Sigma-Aldrich), 100 nM dexamethasone (Sigma-Aldrich), 100 nM humulin R (Lilly, Indianapolis, IN), 1 µM rosiglitazone (Sigma-Aldrich), 0.5 mM 3-isobutyl-1-methylxanthine (Sigma-Aldrich), 2 nM 3,3′,5-triiodo-l-thyronine sodium salt (Sigma-Aldrich) and 10 µg/ml human transferrin (Sigma-Aldrich). Three days after differentiation induction, medium was changed to ASC maintenance medium (ASC-MM) consisting of DMEM:Ham’s F12 1:1, supplemented with 1% penicillin/streptomycin, 33 µM biotin, 17 µM D-pantothenic acid hemicalcium salt, 10 nM dexamethasone and 10 nM humulin R. Cells were cultured at 37 °C, 5% CO_2_.

### Reconstructed human skin

RhS and adipose-RhS were constructed in transwells (0.4 µm, 24 mm; Corning). For the adipose layer, 6 mg/ml collagen, isolated from rat tails and dissolved in 0.1% acetic acid (VWR, Radnor, PA), was mixed with 5 mg/ml fibrinogen (Diagnostica Stago, Paris, France) in a ratio of 1:1. Then, 3.2 × 10^6^ adipocytes were added to 800 µl hydrogel per construct. For fibrin formation and polymerization, 0.5 U/ml thrombin (Merck, Darmstadt, Germany) was added and constructs were incubated at 37 °C, 5% CO_2_ for 90 min. The polymerized adipose layer was submerged in ASC-MM and incubated overnight. Next, the dermis was constructed with 1.4 × 10^5^ dermal fibroblasts in 2 ml hydrogel, composed of 3 mg/ml collagen, 1 mg/ml fibrinogen and 0.5 U/ml thrombin, and incubated overnight. Constructs with adipose layer were submerged in ASC-MM and KCI (1:1) and constructs without adipose layer in KCI. The next day, 5 × 10^5^ KCs were seeded on top and culture medium was supplemented with 2 ng/ml keratinocyte growth factor (Sigma-Aldrich). Skin constructs were cultured submerged for 3 days, at 37 °C, 7.5% CO_2_ from now on, and subsequently cultured at the air–liquid interface in keratinocyte medium II (KCII: DMEM:Ham's F12 3:1, supplemented with 1% penicillin/streptomycin, 1% FetalClone III, 1 µM hydrocortisone, 1 µM isoproterenol hydrochloride, 0.1 µM insulin, 10 µM l-carnitine (Sigma-Aldrich), 10 mM l-serine (Sigma-Aldrich) and 50 μg/ml ascorbic acid (Sigma-Aldrich) or KCII:ASC-MM (1:1), respectively. Cultures were kept at the air–liquid interface for 14 days and incubated in KCII without hydrocortisone 24 h before harvesting. Supernatants were stored at -20 °C until further analysis. Samples were taken for RNA-seq and histology. For RNA-seq, epidermis and dermis were separated, tissue samples were snap frozen in liquid N_2_ and stored at -80 °C. For histology, tissue samples were fixed overnight in 4% formaldehyde (VWR) before preparation for histological analysis.

### RNA sample preparation and sequencing

RNA was isolated using QIAshredder spin columns (Qiagen, Hilden, Germany) and the RNeasy Mini Kit (Qiagen), according to the manufacturer’s instructions. Concentrations and quality (RNA integrity numbers) were measured with a TapeStation (Agilent, Santa Clara, CA). Samples were stored at -80 °C until the library was prepared and sequenced with QuantSeq FWD by Lexogen (Vienna, Austria). Reads were aligned with the Spliced Transcripts Alignment to a Reference (STAR) aligner. For DEG analysis, summary count data was used.

### RNA-sequencing analysis

R (v4.1.2) was used for programming. The R scripts used for analysis can be found at https://github.com/MolecularCellBiologyImmunology/Tri-layered_RhS. Gene counts were normalized and transformed to log count per million (lcpm), low count genes with a lcpm lower than two were removed. After quality control, sample 9 was removed because of too many unmapped reads and the log_2_ fold changes (FCs) were re-shrunk with the method from Zhu, Ibrahim and Love [23]. DESeq2 (v1.34.0) was used to generate principal components (PCs), run principal component analysis (PCA) and identify DEGs. Resulting* p*-values were adjusted according to the number of tests performed and genes were regarded as significantly differentially expressed if* p*_adj_ < 0.05. For GO, Enrichr [24–26] was used with the library WikiPathway_2021_Human.

### Histology, immunohistochemistry and immunofluorescent staining

Confluent adipocyte monolayers were washed with PBS and stained with AdipoRed (Lonza) in PBS (1:33) for 15 min at room temperature (RT) according to the manufacturer’s instructions before imaging. Tissue samples were embedded in paraffin and 5 µm sections were used for morphological (haematoxylin & eosin, H&E) and immunohistochemical stainings. Antibodies for cytokeratin 15 (K15, clone EPR1614Y; Abcam, Cambridge, UK), cytokeratin 10 (K10, clone DE-K10; Progen, Heidelberg, Germany) and vimentin (clone V9; Dako, Glostrup, Denmark) were used as previously described [27, 28]. Prior to embedding in Aquatex (Merck), sections were counter-stained with hematoxylin. For confocal imaging, tissue samples were fixed in 4% formaldehyde overnight, stored in PBS with 0.02% sodium azide (Merck) and subsequently stained with AdipoRed (1:33) and DAPI (1:5000) for 15 min at RT.

### Microscopy

Adipocyte monolayers were imaged using a Nikon ECLIPSE Ti2 (Nikon, Tokyo, Japan). Sections were imaged with a Vectra Polaris slide scanner (Akoya, Marlborough, MA) and analyzed with QuPath (v0.2.2). Confocal images were taken with a Leica TCS SP8 (Leica Microsystems, Wetzlar, Germany) and analyzed using Imaris (v9.9.1; Oxford Instruments, Oxfordshire, UK).

### Quantitative polymerase chain reaction

Adipocyte RNA was isolated with the RNeasy Mini Kit (Qiagen) and reversely transcribed with the RT2 First Strand kit (Qiagen) according to the manufacturer’s instructions. RNA and cDNA concentrations and quality were measured with a NanoPhotometer (Implen, Munich, Germany). The following primers were used (OriGene, Rockville, MD): Krüppel-like factor 15 (KLF15, HP210627), Fatty-acid-binding protein 4 (FABP4, HP205321), Adiponectin (ADIPOQ, HP208060) and Perilipin-1 (PLIN1, HP206315). The reaction mix consisted of Fast SYBR Green Master Mix (Applied Biosystems, Waltham, MA), 300 nM of primer pairs, cDNA and DEPC-Treated Water (Invitrogen, Waltham, MA) with a total volume of 10 µl. Relative changes in mRNA levels were calculated with the 2^−∆∆Ct^ method, using GAPDH as the housekeeping gene, and normalized to expression levels at day 0.

### Enzyme-linked immunosorbent assay and cytokine bead array

Culture supernatant was used to analyze cytokines, IL-6 and IL-8 by ELISA (IL-6: R&D Systems, Minneapolis, MN; IL-8: Diaclone SAS, Besancon, France) according to the manufacturer’s instructions. Cytokine bead arrays: Human Inflammation Panel (BioLegend, San Diego, CA) and Human Adipokine Panel (BioLegend) were used on an Attune NxT flow cytometer (ThermoFisher, Waltham, MA). Data was analyzed with the online available LEGENDplex Data Analysis Software Suite.

### Statistical analysis

**Fig. 1:**
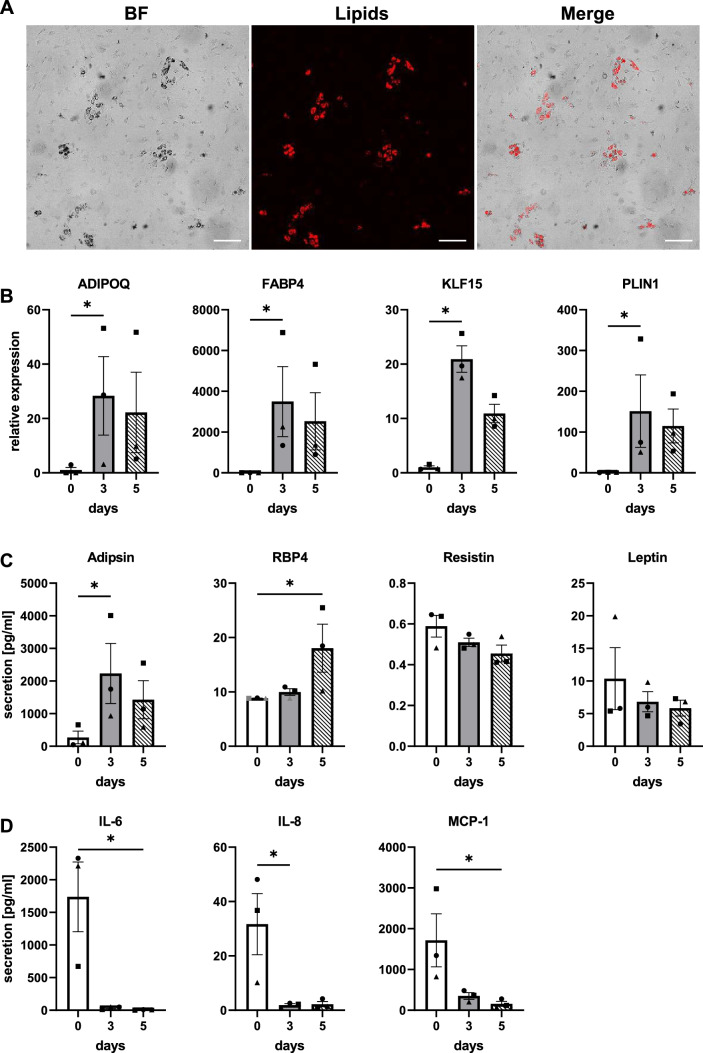
Characterization of adipose-derived stromal cell differentiation to adipocytes in monolayer. **A** Lipid droplet formation in bright field (BF) and with AdipoRed staining on day 7. **B** Gene expression of key adipogenic genes throughout differentiation, relative to housekeeping genes (day 0 was set to 1). **C, D** Secretion of adipokines and pro-inflammatory cytokines into supernatant. ADIPOQ, Adiponectin; FABP4, Fatty-acid-binding protein 4; KLF15, Krüppel-like factor 15; PLIN1, Perilipin-1; RBP4, Retinol binding protein 4; IL, Interleukin; MCP-1, Monocyte chemoattractant protein 1. Scale bar = 200 µm. *p < 0.05. Unconditioned control medium levels as well as gray data points were below detection limit. Shapes represent different donors and bars mean ± SEM; *n* = 3 independent experiments performed in duplicates

**Fig. 2:**
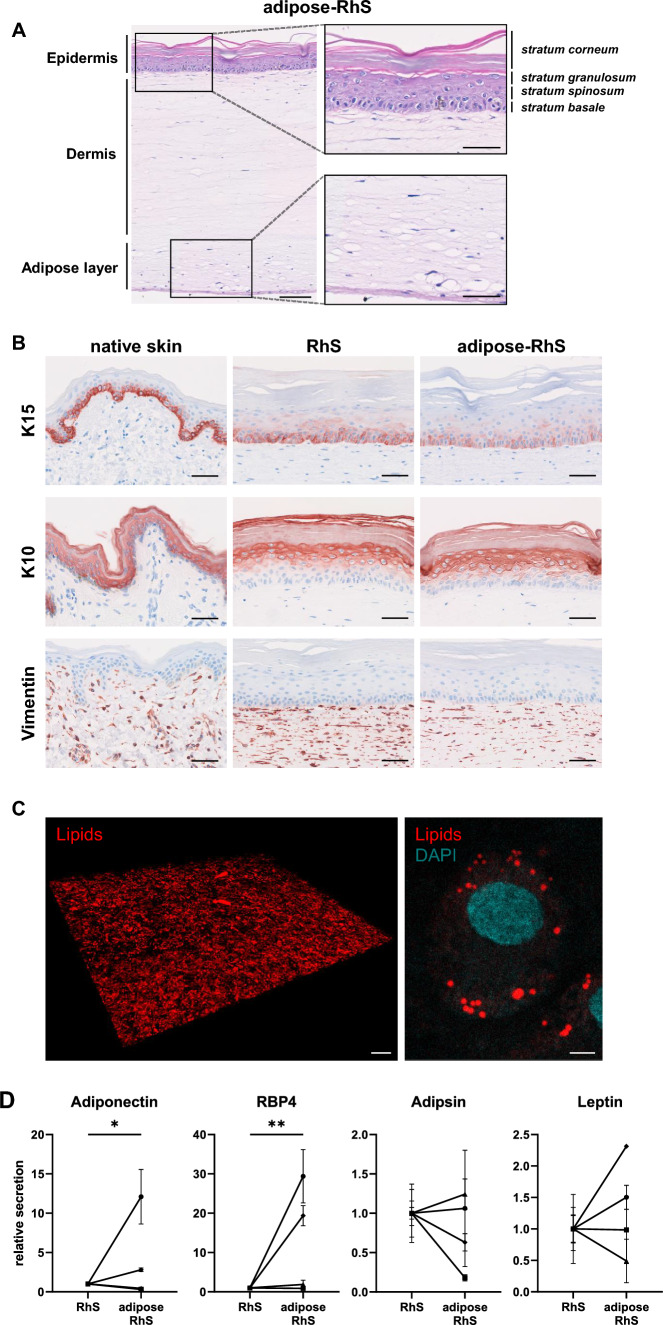
Adipose-RhS characterization by histology and adipokine secretion. **A** H&E staining of the full thickness of adipose-RhS. **B** Comparison of native skin, RhS and adipose-RhS for marker expression of K15, K10 (epidermis) and vimentin (dermis). **C** 3D representation of the adipose layer within adipose-RhS stained for lipids with AdipoRed and nuclei with DAPI. **D** Adipokine secretion into the supernatant of 14-day-old RhS and adipose-RhS. H&E, Hematoxylin & eosin; K, Cytokeratin; RBP4, Retinol binding protein 4. Scale bar = 100 µm in both overviews of A and C, 50 µm in B and magnifications of A and 5 µm in magnification of C. *p < 0.05, **p < 0.01. Representative images from *n* ≤ 4 independent repeats

**Fig. 3:**
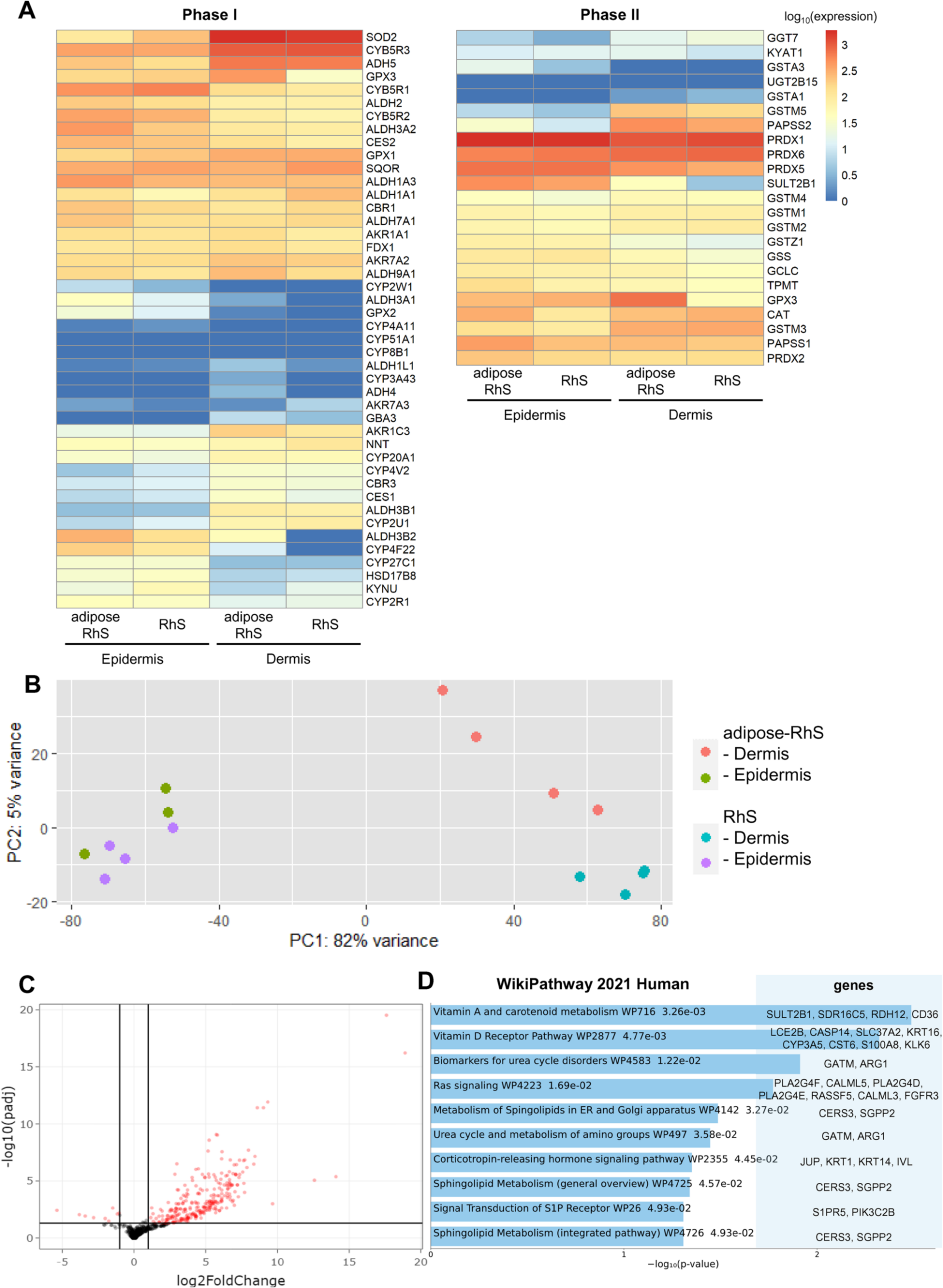
RNA-seq reveals metabolic gene expression in RhS/adipose-RhS and differentially expressed genes and pathways after addition of the adipose layer. **A** Metabolic genes reported to be present (on mRNA, protein or activity level) in *ex vivo* native human skin but not yet described to be present in RhS. **B** Separation of epidermal and dermal samples on PC1 and RhS/adipose-RhS dermis on PC2. **C** Significant differentially expressed genes in red: 295 DEGs were identified, 9 down-regulated and 286 up-regulated. **D** GO analysis with input of 286 up-regulated genes identified in C. Shown are the top 10 pathways, ranked by *p*-value, enriched in the WikiPathway 2021 Human library. Bars contain pathway terms, WikiPathway (WP) identifiers and *p*-values. PC, principal component. n = 4 independent experiments, RNA pooled from intra-experimental triplicates

**Fig. 4:**
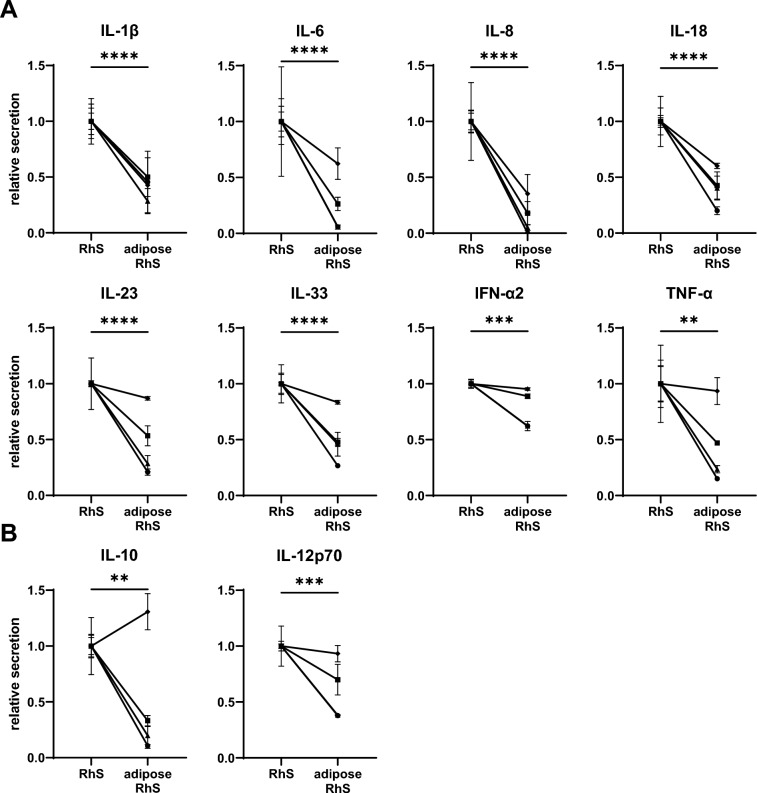
Cytokine secretome of RhS and adipose-RhS. **A, B** Pro-inflammatory and anti-inflammatory cytokines secreted into the medium of both models. Cultures were kept at the air–liquid interface for 14 days. IL, Interleukin; IFN, Interferon; TNF, Tumor necrosis factor. Unconditioned control medium levels were below detection limit. **p < 0.01, ***p < 0.001, ****p < 0.0001. Shapes represent different donors as mean ± SEM; *n* = 4 independent experiments performed in triplicates. Cytokine secretion from adipose-RhS was normalized to mean secretion of RhS of each donor, which was set to 1

Data is presented as mean ± standard error of the mean (SEM). For Fig. 1, three independent repeats, each with different donors of ASCs, were used with two intra-experimental replicates. For RhS experiments (Figs. 2, 3, 4), four independent experiments, each with different donors, were performed with three intra-experimental replicates. KCs, fibroblasts and ASCs all derived from different donors resulting in a total of 12 skin donors were used in the experiments (to minimize donor variation). In graphs, the average from intra-experimental replicates is shown as one data point. GraphPad Prism (v9.1.0; GraphPad Software Inc., La Jolla, CA) was used for statistical analysis. For normality testing, a Shapiro–Wilk test was used and a one-way ANOVA with a Friedman test and a two-way ANOVA. Differences were considered as significant when *p* < 0.05.

## Results

### Adipose derived stromal cells differentiate into a mixed culture containing adipocytes

Prior to constructing RhS, ASCs were cultured under conventional submerged culture conditions and differentiated into adipocytes. Lipid droplet formation, the first visible hallmark of differentiation, was shown by staining with AdipoRed. Adipogenesis started 5 days after initiation of the differentiation regime and became increasingly pronounced until day 7 (Fig. 1A). Expression of a number of key adipogenic genes: ADIPOQ, FABP4, KLF15 and PLIN1 [29–31] increased after 3 days of culture in ASC differentiation medium and remained high for the remaining culture period (Fig. 1B). To verify differentiation on protein level, adipokine secretion into the culture supernatant was measured. Adipsin secretion peaked at differentiation day 3, whereas retinol binding protein 4 (RBP4) increased at day 5 (Fig. 1C). In contrast, resistin and leptin secretion decreased throughout differentiation. Pro-inflammatory cytokine secretion of IL-6 (193-fold) and IL-8 (12-fold) as well as monocyte chemoattractant protein-1 (MCP-1; 11-fold) decreased considerably already after 3 days and stayed at a very low level until day 5, when adipocytes were used to generate the adipose layer (Fig. 1D).

### Histological characterization of RhS and adipose-RhS and adipokine secretion

After monolayer differentiation, adipocytes were incorporated into a hydrogel and the RhS was constructed on top. Histological characterization of adipose-RhS revealed a stratified and differentiated epidermis, a fibroblast-populated hydrogel (dermis) and a hydrogel containing adipocytes (hypodermis; Fig. 2A). The epidermis of both RhS and adipose-RhS consisted of a *stratum corneum*, *stratum granulosum*, *stratum spinosum* and *stratum basale* (Fig. 2A, B). In line with *ex vivo* skin, RhS and adipose-RhS showed K15 expression in undifferentiated KCs within the *stratum basale* and suprabasal K10 expression in differentiating KCs (Fig. 2B). Fibroblasts were present throughout the dermis. Lipid droplets originating from adipocytes within the adipose layer were visualized with an AdipoRed staining (Fig. 2C, left; Supplementary Materials, Movie S1). More than 20 lipid droplets were observed within a single cell (Fig. 2C, right). To assess adipokine production, release into the culture supernatant was measured. Adiponectin and RBP4 were secreted more in adipose-RhS compared to RhS (Fig. 2D). Due to large inter- and intra-experimental variation, no changes could be concluded for secretion of adipsin or leptin.

### Adipose layer changes transcription profiles and activates metabolic pathways

In order to characterize both models and to determine whether or not the adipose-RhS was more metabolically active than RhS, RNA-seq was performed. Epidermis and dermis were analyzed separately to identify DEGs between RhS and adipose-RhS. For simplicity, samples referred to as “dermis” for adipose-RhS contain both the fibroblast-populated hydrogel dermis and the adipocyte hydrogel. Remarkably, independent of the presence of the adipose layer, both RhS and adipose-RhS expressed 36 of 45 phase I and 19 of 23 phase II metabolic genes that have been described to be expressed in *ex vivo* native human skin, but were so far not detected in RhS models described by others (Fig. 3A) [6]. In addition, cultures were characterized in-depth for metabolic gene families described to be expressed by *ex vivo* human skin: cytochrome P450s, alcohol dehydrogenases, aldehyde dehydrogenases, arachidonate lipoxygenases and hydroxysteroid dehydrogenases, sulfotransferases and glutathione s-transferases, of which 79 enzymes were expressed in the described RhS and adipose-RhS (Supplementary Materials, Fig. S1A) [5] out of the 98 enzymes described previously in *ex vivo* human skin, RhS, RhE and KC monolayers (Supplementary Materials, Fig. S1B) [6].

Upon further analysis, the PCA clearly showed separation of epidermal and dermal samples (Fig. 3B). For epidermal samples, it was not possible to distinguish RhS from adipose-RhS transcription profiles, as no individual clusters were visible, indicating that the effect of the adipose layer on the epidermis was very limited (Supplementary Materials, Fig. S2). Therefore, only dermis samples were used for further in-depth analysis. Within the dermis, 9 down- and 286 up-regulated genes were identified in adipose-RhS compared to RhS (Fig. 3C; Supplementary Materials, Tab. S1). Most importantly, among the up-regulated DEGs were genes identified to be involved in phase I metabolism: CYP19A1 (2.4x), CYP4F22 (5.3x), CYP3A5 (6.0x), ALDH3B2 (3.7x), EPHX3 (4.6x) and phase II metabolism: SULT2B1 (2.7x), GPX3 (3.6x; Table 1). GO pathway analysis with the total of 286 up-regulated genes in adipose-RhS revealed that the two most enriched pathways related to metabolism were vitamin A and carotenoid metabolism as well as the vitamin D receptor pathway (Fig. 3D).

**Table 1:**
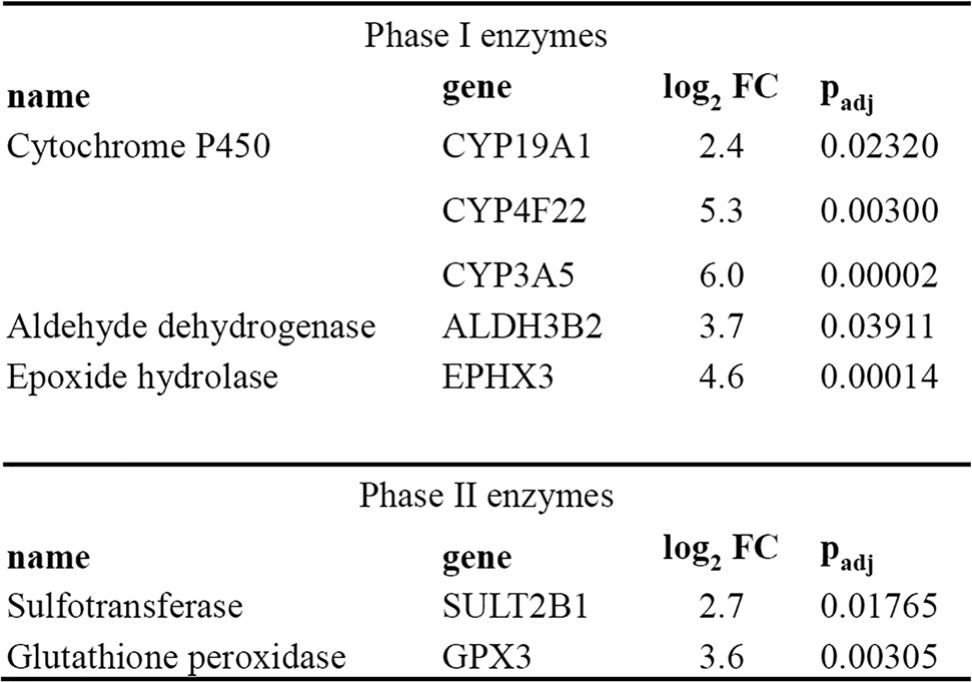
Up-regulated genes in dermal fraction of adipose-RhS involved in phase I and phase II metabolism. FC, fold change

### Reduced inflammatory cytokine secretion in adipose-RhS

Finally, we determined whether the addition of the adipose layer had an effect on the cytokine production of the model. Pro-inflammatory cytokines IL-1β, IL-6, IL-8, IL-18, IL-23, IL-33, interferon alpha 2 (IFN-α2) and tumor necrosis factor alpha (TNF-α) (Fig. 4A) as well as anti-inflammatory cytokines IL-10, IL-12p70 (Fig. 4B) were detected in culture supernatants of the RhS. Notably, upon addition of the adipose layer, decreased secretion of all cytokines was observed with the highest relative reduction being for IL-8 (85%) and IL-6 (78%).

## Discussion

In this study, we show that incorporation of an adipose layer into a RhS results in higher metabolic activity when compared to a RhS with dermis and epidermis only. Of the 295 genes which were differentially expressed, the most enriched pathways were vitamin A and carotenoid as well as the vitamin D receptor pathway. Independently of the presence of an adipose layer, we could confirm expression of 55 metabolic genes within our RhS that have been described to be present in *ex vivo* native human skin but, until now, not in any other *in vitro* skin model [5, 6].

Adipocyte differentiation in adipose-RhS was confirmed by expression of KLF15, ADIPOQ, FABP4 and PLIN1 and secretion of adipsin and RBP4. In humans, two different types of fat exist: white adipose tissue (WAT) and brown adipose tissue (BAT) [32], of which WAT is the most abundant type in subcutaneous tissue [33]. WAT is typically characterized by forming a single large lipid droplet (unilocular) within the cell, whereas BAT typically contains numerous smaller lipid droplets within a cell (multilocular) [34]. As primary ASCs were derived from subcutaneous fat, the differentiated adipocytes and subsequently constructed adipose layer within adipose-RhS would be expected to be WAT. However, the differentiated adipocytes were multilocular and therefore incorporated small lipid droplets. This could be due to the ASC-derived adipocytes not being fully mature, as also described in studies by others [35, 36]. Notably, when ASC-derived adipocytes were maintained under conventional submerged conditions in differentiation medium for 28 days, more as well as bigger lipid droplets were observed within the cells (data not shown). Therefore, it may be possible to increase the volume of intracellular lipids and achieve unilocularity in the future by extending the culture periods used in this current study.

It has been shown that medium composition is key in maintaining intact lipids and wrong culture medium can even lead to de-differentiation of mature adipocytes [37]. To counteract this issue and further mature ASCs inside the model throughout the culture period, the deliberate choice to use different media for RhS and adipose-RhS has been made. This could also result in a limitation of this study as different culture media could influence gene expression and cytokine secretion. Main components added to adipose-RhS when also cultured in ASC-MM medium are d-pantothenic acid, biotin and dexamethasone. To our knowledge, d-pantothenic acid and biotin have no immunomodulatory effects on fibroblasts or KCs, neither do they have any metabolic effects on these cell types. In contrast, dexamethasone is a well characterized anti-inflammatory drug. It has been reported that dexamethasone incubation prior to KC lysate exposure (cultured under conventional submerged conditions) was able to suppress secretion of IL-6 and IL-8 in dermal fibroblasts [38]. However, a 200-fold higher concentration of dexamethasone was used and, in contrast to our study, the cells were in direct contact with the dexamethasone rather than being exposed to it in an air–liquid transwell culture system where the effective concentration is lower than that of the culture medium. Therefore, it is most unlikely that our results were influenced by different medium composition between RhS and adipose-RhS.

The ASC differentiation does not result in 100% adipocytes but a mix of adipocytes and stromal cells [20]. Pro-inflammatory and anti-inflammatory cytokine secretion decreased considerably in adipose-RhS (and differentiated adipocyte monocultures). This is in line with previous studies conducted with an ASC-derived hypertrophic scar model and indicates that the addition of the adipose layer containing a mixture of stromal cells and adipocytes leads to a lower inflammatory state and contributes to tissue homeostasis [39]. It is also generally known that ASCs have immune-regulatory effects [40, 41]. Other findings confirmed that ASCs can contribute to epidermal homeostasis, e.g. a co-culture of RhE with ASCs resulted in epidermal thickening and a KC-ASC co-culture increased the proliferative and adhesive potential of KCs [42].

The aim of our study was to determine how the adipose layer changes metabolic enzyme expression within RhS. Because of technical reasons, the dermis and adipose layer were sequenced together and therefore it cannot be determined whether gene up- and down-regulation was directly due to the adipose layer or whether it was an indirect effect of adipocytes increasing metabolic enzyme expression in fibroblasts. In the future, this can be addressed by either separation of the two layers or by single-cell sequencing.

Nevertheless, clear differences were observed in the transcriptomic profile of adipose RhS (dermis) with 7 phase I and phase II enzymes being identified, all of which were higher expressed in adipose-RhS. The cytochrome P450 3A5 (CYP3A5) and 4F22 were up-regulated the most. Even though expressed at a much lower level compared to the liver, presence as well as activity of CYP3A5 have been described earlier in native skin and a commercial RhS (EpiDerm- FT400™ TESE) [43]. Strikingly, in a screen of the 200 most prescribed drugs in the U.S., the CYP3A4/5 subfamily was responsible for the metabolism of 37% of the drugs which were hepatically cleared. Out of 7 subfamilies: cytochrome 3A, 2E1, 2D6, 2C19, 2C9, 2B6 and 1A, CYP3A4/5 made up the largest part of CYP-mediated clearance [44, 45]. CYP19A1, epoxide hydrolase 3 (EPHX3) and glutathione peroxidase (GPX3) have not been described to be present in RhS before. However, they were up-regulated in our adipose-RhS model. CYP19A1 (human aromatase) is a key enzyme in estrogen biosynthesis and was up-regulated as well. It catalyzes the reaction from testosterone to estradiol and is known to be expressed in adipose tissue [46]. Gene expression of the phase I enzyme EPHX3 was also up-regulated and it has been suggested that EPHX3 plays a primary role in xenobiotic metabolism in the liver [47]. Furthermore, EPHX3 deficient mice show impaired skin barrier function [48]. GPX3 has been identified by proteomic profiling to be present in *ex vivo* native skin but not in 2D KC cultures, RhE or conventional two-layered RhS until now [49]. Of note, all genes mentioned above were differentially expressed without metabolic stimulation. Therefore, future studies should focus on changes in metabolism in adipose-RhS/RhS after e.g. exposure to irritants or sensitizers.

Taken together, up-regulation and function of these enzymes emphasize their importance and show the added value of the adipose layer in the RhS. GO analysis of the up-regulated genes in the dermis reveals that the most enriched pathways in adipose-RhS were vitamin A and carotenoid metabolism as well as the vitamin D receptor pathway. Vitamin D is synthesized in human skin, its metabolism is well studied and described and deficiencies can result in dermatological conditions such as psoriasis and atopic dermatitis [50–52]. It has several immune-regulatory properties, has anti-bacterial, anti-viral and anti-inflammatory functions [53]. This is in line with the measured decrease in the secretion of pro-inflammatory cytokines in the adipose-RhS model. Assessing the secretome of both models, a reduction for all pro-inflammatory cytokines (IL-1β, IL-6, IL-8, IL-18, IL-23, IL-33, IFN-α2 and TNF-α) was observed when the adipose layer was incorporated.

Similar to vitamin D, vitamin A and its active metabolite retinoic acid play an important role in skin where they are also involved in tissue homeostasis and orchestrate the immune responses [54]. Retinoids are commonly used to treat psoriasis, ichytosis, acne and wrinkles [55, 56]. They can act pro-inflammatory in some microenvironments but mainly function in an anti-inflammatory fashion [57].

In summary, we describe the construction and comprehensive characterization of a RhS model with an adipose layer. Our findings indicate that the adipose layer lowers the inflammatory state of the RhS model, is beneficial for tissue homeostasis, increases metabolic activity and possesses a similar histology compared to native skin. This model is the first step towards more physiological skin models to study human skin in health and disease. In addition, it can be used as a valuable tool for risk assessment of actives which are exposed to skin. Further research should focus on xenobiotic exposure of the adipose-RhS as well as the incorporation of immune cells, appendages such as hair follicles [58] and vasculature or its combination with other organs in microphysiological systems (MPS; multi-organ-chips) [59, 60]. Combining recent advances in MPS, bioprinting as well as induced pluripotent stem cells offers exciting new possibilities to create more complex skin models and recapitulate human skin in health and disease more closely in the future.

### Supplementary Information

Below is the link to the electronic supplementary material.

Supplementary Movie S1: Overview of adipose layer within RhS. Lipids were stained with AdipoRed. (MP4 66883 KB)

Supplementary Fig. S1 and S2 (PPTX 886 KB)

Supplementary Tab. S1: List of up- and down-regulated genes in adipose-RhS dermal samples. (XLSX 38 KB)

## Data Availability

The data discussed in this publication have been deposited in NCBI's Gene Expression Omnibus and are accessible through GEO Series accession number GSE232638 (https://www.ncbi.nlm.nih.gov/geo/query/acc.cgi?acc=GSE232638).

## References

[1] Mukhtar H, Bickers DR. Drug metabolism in skin. Comparative activity of the mixed-function oxidases, epoxide hydratase, and glutathione S-transferase in liver and skin of the neonatal rat. Drug Metab Dispos. 1981;9:311–4. 6114828

[2] Baron JM, Merk HF (2001). Drug metabolism in the skin. Curr Opin Allergy Clin Immunol..

[3] Cibrian D, de la Fuente H, Sánchez-Madrid F (2020). Metabolic pathways that control skin homeostasis and inflammation. Trends Mol Med.

[4] Taylor NJ (2020). Metabolomics of primary cutaneous melanoma and matched adjacent extratumoral microenvironment. PLoS ONE.

[5] Gibbs S (2007). Xenobiotic metabolism in human skin and 3D human skin reconstructs: a review. Curr Drug Metab.

[6] Kazem S, Linssen EC, Gibbs S (2019). Skin metabolism phase I and phase II enzymes in native and reconstructed human skin: a short review. Drug Discovery Today.

[7] Hartung T (2008). Food for thought... on animal tests. ALTEX..

[8] Alépée N (2010). A catch-up validation study on reconstructed human epidermis (SkinEthic™ RHE) for full replacement of the Draize skin irritation test. Toxicol In Vitro.

[9] Gibbs S (2013). An epidermal equivalent assay for identification and ranking potency of contact sensitizers. Toxicol Appl Pharmacol.

[10] Desprez B, Barroso J, Griesinger C, Kandárová H, Alépée N, Fuchs HW. Two novel prediction models improve predictions of skin corrosive sub-categories by test methods of OECD Test Guideline No. 431. Toxicol In Vitro. 2015;29:2055–80. 10.1016/j.tiv.2015.08.015 26320836

[11] Kosten IJ (2015). MUTZ-3 derived Langerhans cells in human skin equivalents show differential migration and phenotypic plasticity after allergen or irritant exposure. Toxicol Appl Pharmacol.

[12] Pellevoisin C (2021). Pre-validation of SENS-IS assay for in vitro skin sensitization of medical devices. Toxicol In Vitro.

[13] Rivera-Gonzalez G, Shook B, Horsley V (2014). Adipocytes in skin health and disease. Cold Spring Harb Perspect Med..

[14] Trottier V (2008). IFATS collection: using human adipose-derived stem/stromal cells for the production of new skin substitutes. Stem Cells.

[15] Bellas E (2012). In vitro 3D full-thickness skin-equivalent tissue model using silk and collagen biomaterials. Macromol Biosci.

[16] Monfort A (2012). Production of human tissue-engineered skin trilayer on a plasma-based hypodermis. J Tissue Eng Regen Med.

[17] Kober J (2015). Generation of a fibrin based three-layered skin substitute. Biomed Res Int.

[18] Kim BS (2019). 3D ell printing of perfusable vascularized human skin equivalent composed of epidermis, dermis, and hypodermis for better structural recapitulation of native skin. Adv Healthc Mater.

[19] Zimoch J (2021). Bio-engineering a prevascularized human tri-layered skin substitute containing a hypodermis. Acta Biomater.

[20] Kroeze KL (2009). Chemokine-mediated migration of skin-derived stem cells: Predominant role for CCL5/RANTES. J Investig Dermatol.

[21] Waaijman T (2010). Use of a collagen-elastin matrix as transport carrier system to transfer proliferating epidermal cells to human dermis in vitro. Cell Transplant.

[22] Lee MJ, Fried SK (2014). Optimal protocol for the differentiation and metabolic analysis of human adipose stromal cells. Methods Enzymol..

[23] Zhu A, Ibrahim JG, Love MI (2019). Heavy-tailed prior distributions for sequence count data: removing the noise and preserving large differences. Bioinformatics.

[24] Chen EY (2013). Enrichr: interactive and collaborative HTML5 gene list enrichment analysis tool. BMC Bioinformatics.

[25] Kuleshov MV (2016). Enrichr: a comprehensive gene set enrichment analysis web server 2016 update. Nucleic Acids Res.

[26] Xie Z (2021). Gene set knowledge discovery with enrichr. Curr Protocols.

[27] Vahav I (2020). Reconstructed human skin shows epidermal invagination towards integrated neopapillae indicating early hair follicle formation in vitro. J Tissue Eng Regen Med.

[28] Vriens AP (2008). Comparison of autologous full-thickness gingiva and skin substitutes for wound healing. Cell Transplant.

[29] Wu Z, Wang S (2013). Role of kruppel-like transcription factors in adipogenesis. Dev Biol.

[30] Ullah M (2013). Reverse differentiation as a gene filtering tool in genome expression profiling of adipogenesis for fat marker gene selection and their analysis. PLoS ONE.

[31] Ambele MA (2016). Genome-wide analysis of gene expression during adipogenesis in human adipose-derived stromal cells reveals novel patterns of gene expression during adipocyte differentiation. Stem Cell Res.

[32] Rosen ED, Spiegelman BM (2014). What we talk about when we talk about fat. Cell.

[33] Zwick RK (2018). Anatomical, physiological, and functional diversity of adipose tissue. Cell Metab.

[34] Cannon B, Nedergaard JAN (2004). Brown adipose tissue: function and physiological significance. Physiol Rev.

[35] Volz A-C (2019). Comparing the use of differentiated adipose-derived stem cells and mature adipocytes to model adipose tissue in vitro. Differentiation.

[36] Bahmad HF (2020). Modeling adipogenesis: current and future perspective. Cells.

[37] Huber B, Kluger PJ (2015). Decelerating mature adipocyte dedifferentiation by media composition. Tissue Eng Part C Methods.

[38] Murakami S (2022). An epidermal keratinocyte homogenate induced type 2 and proinflammatory cytokine expression in cultured dermal cells. J Dermatol Sci.

[39] van den Broek LJ (2012). Development, validation and testing of a human tissue engineered hypertrophic scar model. ALTEX.

[40] Sakers A (2022). Adipose-tissue plasticity in health and disease. Cell.

[41] Al-Ghadban S, Bunnell BA (2020). Adipose tissue-derived stem cells: immunomodulatory effects and therapeutic potential. Physiology.

[42] Moriyama M (2019). Adipose-derived stromal/stem cells improve epidermal homeostasis. Sci Rep.

[43] Smith SA (2018). Expression and enzyme activity of cytochrome P450 enzymes CYP3A4 and CYP3A5 in human skin and tissue-engineered skin equivalents. Exp Dermatol.

[44] Zanger UM (2008). Functional pharmacogenetics/genomics of human cytochromes P450 involved in drug biotransformation. Anal Bioanal Chem.

[45] Wienkers LC, Heath TG (2005). Predicting in vivo drug interactions from in vitro drug discovery data. Nat Rev Drug Discovery.

[46] Simpson ER (1992). Regulation of human aromatase cytochrome P450 gene expression. J Steroid Biochem Mol Biol.

[47] Marowsky A, Arand M. Mammalian Epoxide Hydrolases. In: Comprehensive Toxicology : Third Edition. Oxford: Elsevier, pp. 308–325.

[48] Edin ML (2021). Epoxide hydrolase 3 (Ephx3) gene disruption reduces ceramide linoleate epoxide hydrolysis and impairs skin barrier function. J Biol Chem.

[49] van Eijl S (2012). Elucidation of xenobiotic metabolism pathways in human skin and human skin models by proteomic profiling. PLoS ONE.

[50] Kechichian E, Ezzedine K (2018). Vitamin D and the skin: an update for dermatologists. Am J Clin Dermatol.

[51] Umar M (2018). Vitamin D and the pathophysiology of inflammatory skin diseases. Skin Pharmacol Physiol.

[52] Saponaro F, Saba A, Zucchi R (2020). An Update on Vitamin D metabolism. Int J Molec Sci..

[53] Bishop E, Ismailova A, Dimeloe S, Hewison M, White JH (2021). Vitamin D and immune regulation: antibacterial, antiviral, anti-Inflammatory. JBMR Plus..

[54] Mora JR, Iwata M, von Andrian UH (2008). Vitamin effects on the immune system: vitamins A and D take centre stage. Nat Rev Immunol.

[55] Lee D-D (2009). Retinoid-responsive transcriptional changes in epidermal keratinocytes. J Cell Physiol.

[56] Szymański Ł, Skopek R, Palusińska M, Schenk T, Stengel S, Lewicki S (2020). Retinoic acid and its derivatives in skin. Cells.

[57] Erkelens MN, Mebius RE (2017). Retinoic acid and immune homeostasis: a balancing act. Trends Immunol.

[58] Abaci HE (2018). Tissue engineering of human hair follicles using a biomimetic developmental approach. Nat Commun.

[59] Kühnl J (2021). Characterization of application scenario-dependent pharmacokinetics and pharmacodynamic properties of permethrin and hyperforin in a dynamic skin and liver multi-organ-chip model. Toxicology.

[60] Tao TP, Brandmair K, Gerlach S, Przibilla J, Géniès C, Jacques-Jamin C (2021). Demonstration of the first-pass metabolism in the skin of the hair dye, 4-amino-2-hydroxytoluene, using the Chip2 skin–liver microphysiological model. J Appl Toxicol..

