# Supplementary material for: Reconstructed Human Skin with Hypodermis Shows Essential Role of Adipose Tissue in Skin Metabolism
Source: Tissue Eng Regen Med. 2024 Feb 17;21(3):499–511. doi: 10.1007/s13770-023-00621-1 (PMC10987437; doi:10.1007/s13770-023-00621-1)
Supplement: Supplementary file 2 — Supplementary Fig. S1 and S2 (PPTX 886 KB) [file 13770_2023_621_MOESM2_ESM.pptx]

## Slide 1
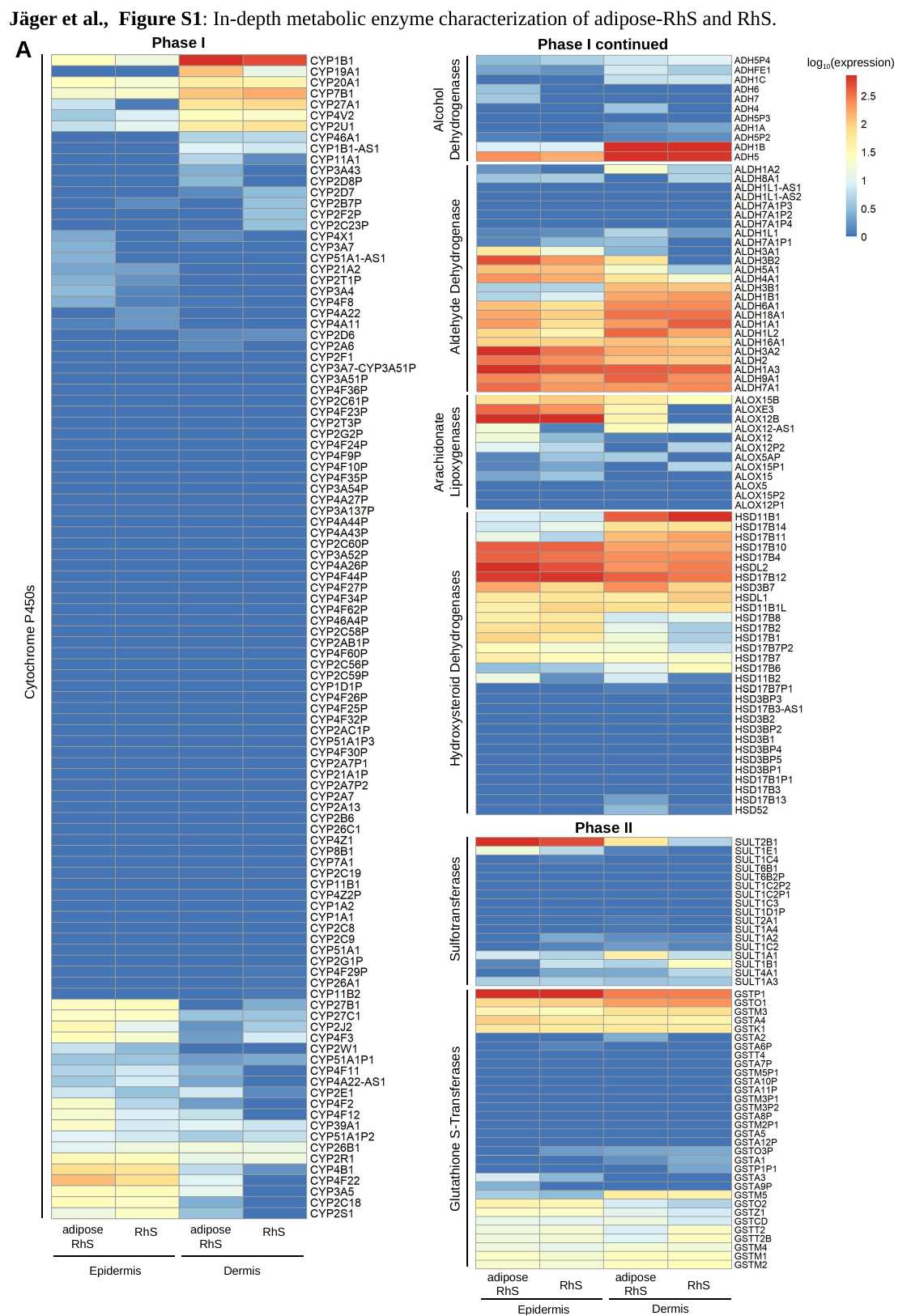

Jäger et al., Figure S1: In-depth metabolic enzyme characterization of adipose-RhS and RhS.
Phase I
Phase I continued
A
log10(expression)
Alcohol
Dehydrogenases
Aldehyde Dehydrogenase
Arachidonate
Lipoxygenases
Cytochrome P450s
Hydroxysteroid Dehydrogenases
Phase II
Sulfotransferases
Glutathione S-Transferases
adipose
RhS
adipose
RhS
RhS
RhS
Epidermis
Dermis
adipose
RhS
adipose
RhS
RhS
RhS
Dermis
Epidermis

## Slide 2
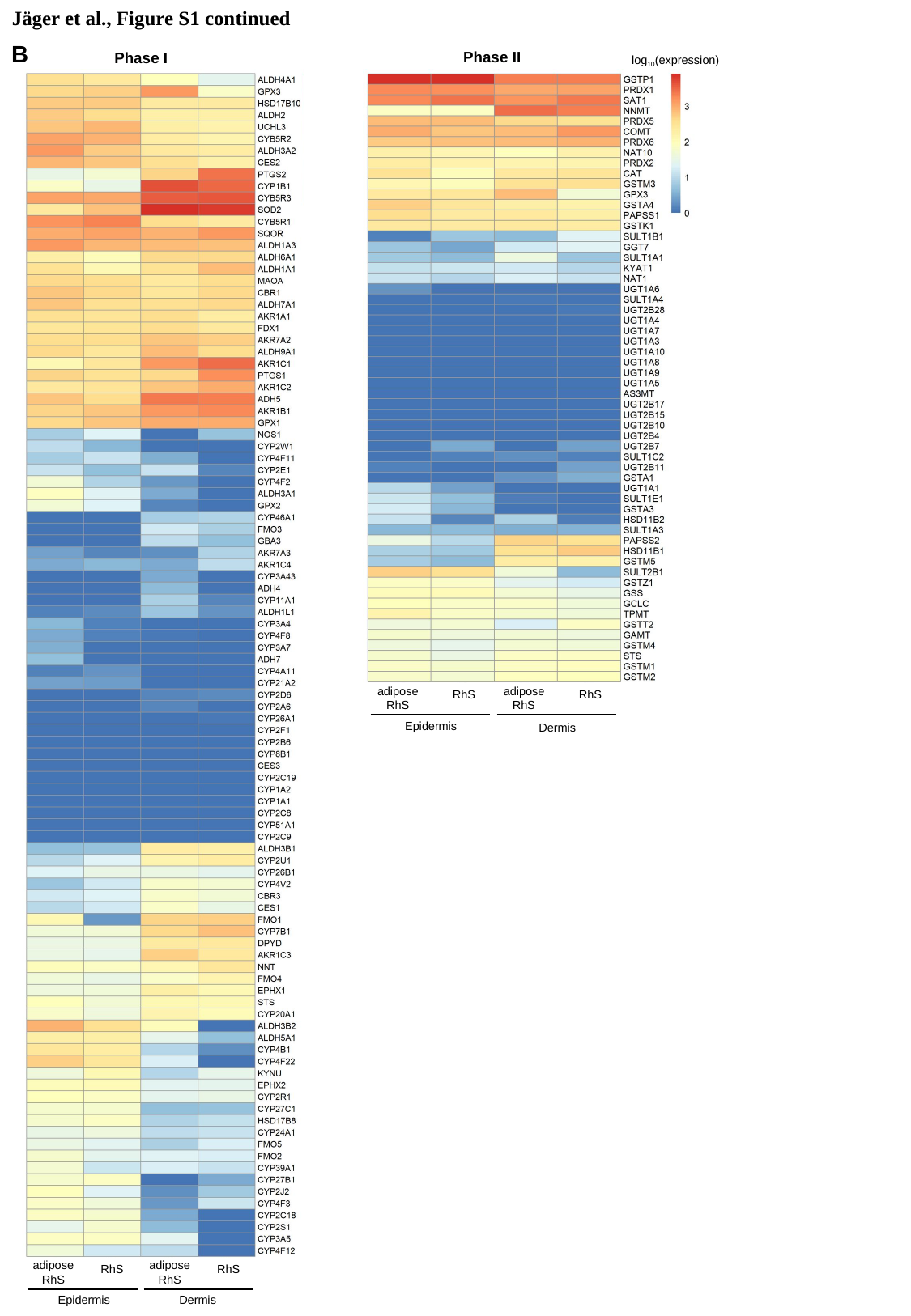

Jäger et al., Figure S1 continued
B
Phase II
Phase I
log10(expression)
adipose
RhS
adipose
RhS
RhS
RhS
Epidermis
Dermis
adipose
RhS
adipose
RhS
RhS
RhS
Dermis
Epidermis

## Slide 3
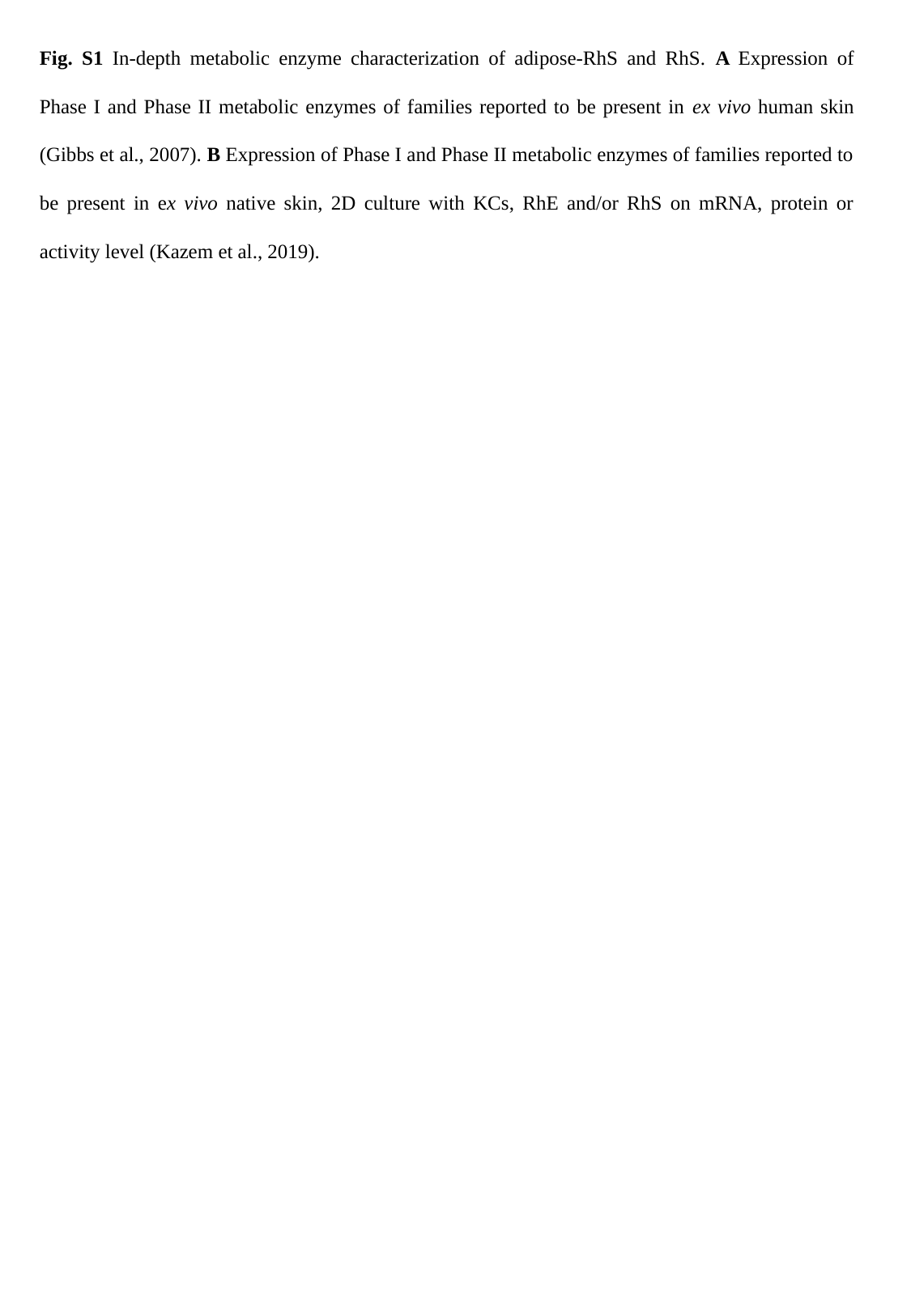

Fig. S1 In-depth metabolic enzyme characterization of adipose-RhS and RhS. A Expression of Phase I and Phase II metabolic enzymes of families reported to be present in ex vivo human skin (Gibbs et al., 2007). B Expression of Phase I and Phase II metabolic enzymes of families reported to be present in ex vivo native skin, 2D culture with KCs, RhE and/or RhS on mRNA, protein or activity level (Kazem et al., 2019).

## Slide 4
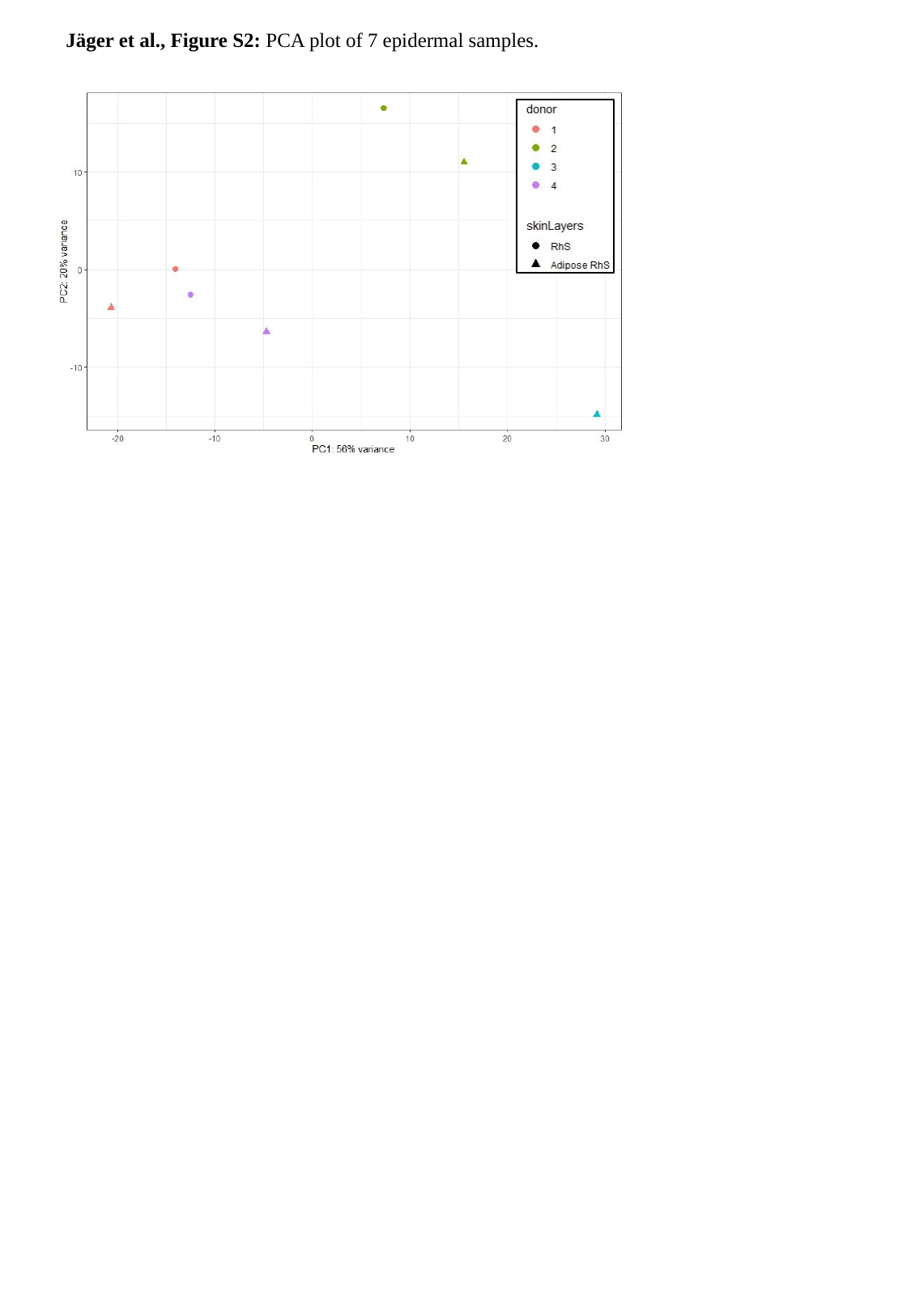

Jäger et al., Figure S2: PCA plot of 7 epidermal samples.

## Slide 5
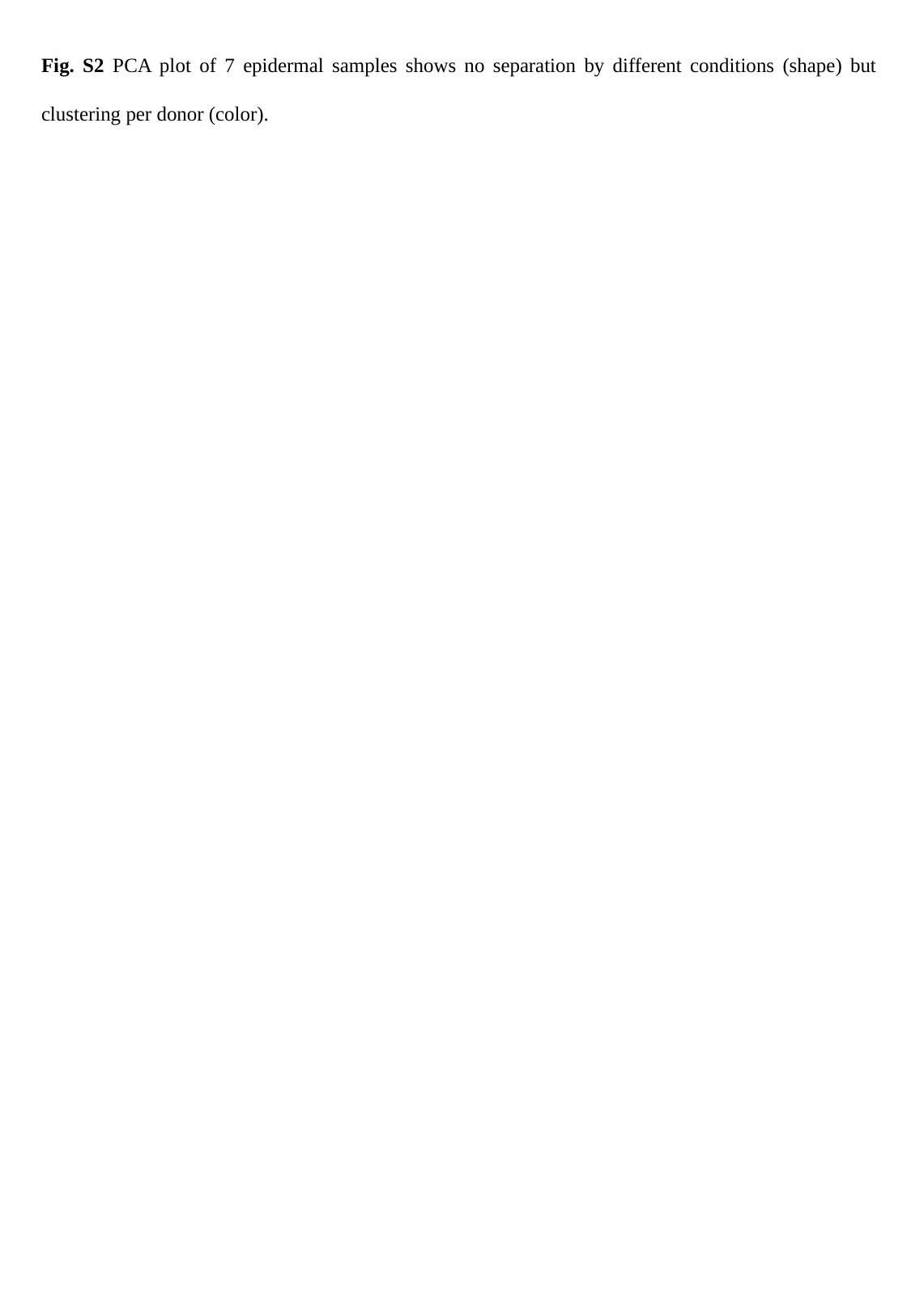

Fig. S2 PCA plot of 7 epidermal samples shows no separation by different conditions (shape) but clustering per donor (color).
